# Quantitative Correlation between the Degree of Reaction and Compressive Strength of Metakaolin-Based Geopolymers

**DOI:** 10.3390/ma13245784

**Published:** 2020-12-18

**Authors:** Xu Chen, Eric Kim, Prannoy Suraneni, Leslie Struble

**Affiliations:** 1 Department of Civil, Environmental, and Architectural Engineering, University of Colorado Boulder, 1111 Engineering Drive UCB 428, Boulder, CO 80309, USA; 2 ExxonMobil Research & Engineering Company, North American Growth Venture, Olefins Furnaces, 3525 Decker Drive, Baytown, TX 77520, USA; eric.kim@exxonmobil.com; 3 Department of Civil, Architectural and Environmental Engineering, University of Miami, Coral Gables, FL 33146, USA; suranenip@miami.edu; 4 Department of Civil and Environmental Engineering, University of Illinois at Urbana Champaign, 205 N. Mathews Ave, Urbana, IL 61801 (emeritus), USA; lstruble@illinois.edu

**Keywords:** geopolymer, nuclear magnetic resonance, quantification, compressive strength

## Abstract

For geopolymers (usually composed of unreacted precursor and gel), the compressive strength is controlled by two factors. The first is the degree of reaction, or, equivalently, the amount of gel formed, including any calcium silicate hydrate gel in calcium-containing mixtures. The second factor is the gel composition, generally given by the Si/Al ratio. These two parameters are interrelated for typical silicate-activated metakaolin geopolymers. By separating out effects of Si/Al ratio and degree of reaction, this study quantitatively correlates the degree of reaction with the compressive strength of metakaolin-based geopolymers with and without calcium. Solid-state ^29^Si nuclear magnetic resonance (NMR) aided with chemical extractions was used to determine gel amounts and composition for several geopolymer mixtures. The compressive strength was also measured for each mixture at 7 days. Both the increase of Na/Al ratio in mixtures without calcium and addition of external calcium increased the degree of reaction, and compressive strength correlated linearly (R^2^ > 0.88) with the degree of reaction.

## 1. Introduction

Geopolymers are synthesized by activating aluminosilicate precursors with sodium/potassium hydroxides and/or silicates. Geopolymers have attracted significant research interest [1,2,3] in the last two decades. They are, in principle, environmentally friendly and low-emission alternatives to conventional portland cement, as synthesis can be carried out at room temperature using industrial wastes such as fly ash, slag, incineration bottom ash, and red mud [2,4,5]. They can exhibit several attractive and exploitable properties, including high compressive strength, low shrinkage, a wide range of setting times, high thermal stability, acid resistance, and a potential to immobilize toxic metals [1,2,4,6,7,8,9].

Metakaolin is a simple aluminosilicate precursor material that facilitates understanding of the composition–structure–property relationships for geopolymers. Unlike fly ashes, it is almost entirely amorphous and reacts relatively rapidly [10]. During geopolymerization, metakaolin is dissolved in an alkaline or alkali-silicate solution to release silicon (Si) and aluminum (Al) ions, which then condense with each other to form aluminosilicate oligomers, building blocks that further condense to form the geopolymer gel [2,11]. Also unlike fly ashes and slags, metakaolin geopolymers do not contain any calcium. For this reason, metakaolin geopolymers provide a convenient system for systematically studying the influence of calcium on the composition and structure of geopolymers and on the properties of geopolymer mixtures.

In the absence of calcium, it seems reasonable that the amount of geopolymer gel will be the parameter most directly influencing the compressive strength (referred to as strength from this point forward) development. It is also known that higher Si/Al ratio in the geopolymer gel reduces strength [12]. In geopolymers activated by solutions containing Si in addition to alkali (e.g., Na^+^ and K^+^), such as used in the current study, reaction is slow and Si/Al ratio in the gel is extremely high, both likely to result in low strength. While both parameters have been studied in detail, a study of the literature reveals that the effects of the degree of reaction and Si/Al ratio on the strength have so far not been separately determined. Such separated understanding is critical, especially when exploring other potential sustainable precursors (e.g., aluminosilicate wastes/minerals with a wide range of compositions and reactivities) that would alter the degree of reaction and Si/Al ratio of the gel so as to produce different strength responses.

To control the amount of the gel independently of its Si/Al ratio, the amount of alkali cations in the solution can be adjusted when designing the mixtures. During gel formation, the alkali cations balance the charges due to Al incorporation [13]. An insufficient amount of alkali prevents complete incorporation of Al into the geopolymer framework (i.e., reduces the formation of geopolymer gel). The incorporation of Al increases with the ratio of M/Al (where M is Na or K) until it reaches a value of 1.0. By adjusting the amount of alkali cations, the degree of reaction can be tailored to determine its correlation with strength.

In the presence of calcium, calcium aluminosilicate hydrate (C-A-S-H) is present in addition to the geopolymer gel and unreacted precursor [14,15,16,17], though calcium ions have also been shown recently to occupy the charge-balancing sites in the structure of geopolymer gel [18]. Unlike the two-dimensional chain-like calcium silicate hydrate (C-S-H) or the somewhat more polymerized but still essentially chain-like C-A-S-H, the structure of geopolymer gel in the presence of Ca is a three-dimensional network [19]. The degree of reaction (equivalently, the amount of C-A-S-H and geopolymer gel relative to the entire mixture) can be reasonably expected to affect strength, but this effect has not been studied with varying amounts of Ca. In a previous study, the higher strength due to the addition of Ca was tentatively attributed to filling of voids within the geopolymer gel by C-A-S-H gel [20]. In another study [21], decreased strength in Ca-geopolymers cured at elevated temperature was attributed to insufficient development of three-dimensional geopolymer gel, while increased strength of the same mixtures cured at room temperature was attributed to enhanced dissolution of the precursor and the additional precipitation of C-A-S-H. Such increased strength with calcium was also reported for fly ash/slag geopolymers in a recent study [22]. However, neither study quantified the degree of reaction in their mixtures. In our previous study of metakaolin geopolymers before and around setting [15], we demonstrated that dissolution of metakaolin and setting of the mixtures were enhanced by calcium hydroxide (Ca(OH)_2_). Here we hypothesize that the addition of calcium also promotes the degree of reaction at later ages and further increases the strength.

The objectives of this study are to quantitatively correlate the degree of reaction with the strength for metakaolin geopolymers with and without calcium. Though a generally linear correlation between degree of reaction and strength has been well established for ordinary portland cements [23,24] and some attempts have been made to understand the strength as a function of multiple mixture parameters in geopolymers [25,26], the current study is the first to correlate strength with degree of reaction independently of other confounding factors. The mixtures were designed such that the effects of degree of reaction could be considered independently of the gel Si/Al ratio. The degree of reaction and composition of the gel were characterized using solid-state ^29^Si NMR spectroscopy [15,27]. The NMR spectra shown here were presented in the thesis of the second author [28] but were reanalyzed here to quantify the degree of reaction. The quantification was validated by chemical extractions [15]. The correlation of degree of reaction with strength provides guidance at a fundamental level to design mixtures with optimized mechanical performance. The effects of calcium on the degree of reaction provide insight into Ca-containing geopolymers—the reaction products and their structures—including hybrid geopolymer–portland cements.

## 2. Materials and Methods

To design geopolymer mixtures with different reaction extent and gel composition, Na/Al ratios and calcium contents were varied as summarized in Table 1. Mixtures 1–4 do not contain any Ca. By varying the Na/Al ratios, specimens with different amounts of gel (corresponding to differing degree of reaction) were synthesized. The Si/Al ratios of these mixtures varied from 1.1 to 1.5, a range much narrower than that studied by Williams et al. [29] so as to minimize the effects of the gel Si/Al ratio on mechanical strength. Different amounts of Ca were added in Mixtures 4–7, with Ca/Si ratios up to 0.15.

Geopolymer mixtures were synthesized using metakaolin (MetaMax^®^, BASF, Ludwigshafen, Germany) as the precursor. The average particle size of metakaolin is 1.3 µm based on the manufacturer’s data. The XRD and XRF results shown separately [28] indicate that the metakaolin is amorphous and contains around 44 wt % of Al_2_O_3_ and 53 wt % of SiO_2_. The activating solution was prepared using a sodium silicate solution (29.02 wt % SiO_2_, 9.00 wt % Na_2_O and 61.98 wt % H_2_O, Fisher, Waltham, MA, USA) and reagent-grade sodium hydroxide (Fisher, Waltham, MA, USA).

During mixing, the activating solution and the metakaolin were first stored at room temperature (~23 °C) for 24 h. For mixtures containing Ca, the desired amount of calcium hydroxide was first mixed with the metakaolin precursor. The precursor (with or without calcium hydroxide) was then mixed with the activating solution in a Hobart^TM^ (N50) paddle mixer using the following protocol: mixed at a low speed for 2.5 min, then stopped for 1.0 min and scraped paste off the sides of the bowl, and finally mixed for another 2.5 min at a high speed.

Immediately after mixing, specimens were placed in two layers in plastic cube molds, 50 × 50 × 50 mm. Each specimen was then vibrated for around 30 s to achieve adequate consolidation. Specimens were stored in a curing room at 25 °C and 100% relative humidity for around 3 h and then heated at 60 °C and ambient humidity and pressure for 2 h. After this heat treatment, they were put back in the curing room until around 24 h after mixing, when they were demolded. The demolded specimens were kept in the curing room until testing.

All tests were carried out at 7 days after mixing. Compressive strengths were measured in accordance with ASTM C109 [30] using a Forney^TM^ compression testing machine with a loading rate of 900–1800 N/s at ambient temperature (22 ± 1 °C). In preparation for ^29^Si NMR testing, samples were ground and then solvent-exchanged to remove water and thereby stop reaction [31].

Solid-state ^29^Si NMR tests were conducted using a Varian Unity Inova spectrometer with magnetic field of 7.04 T at a resonance frequency of 59.6 MHz and a 4-mm probe. Recycle delay and number of scans were 30 s and 2048, respectively. The amount of the geopolymer gel and its Si/Al ratio were calculated based on intensities of the deconvoluted peaks in the spectra.

**Figure 1:**
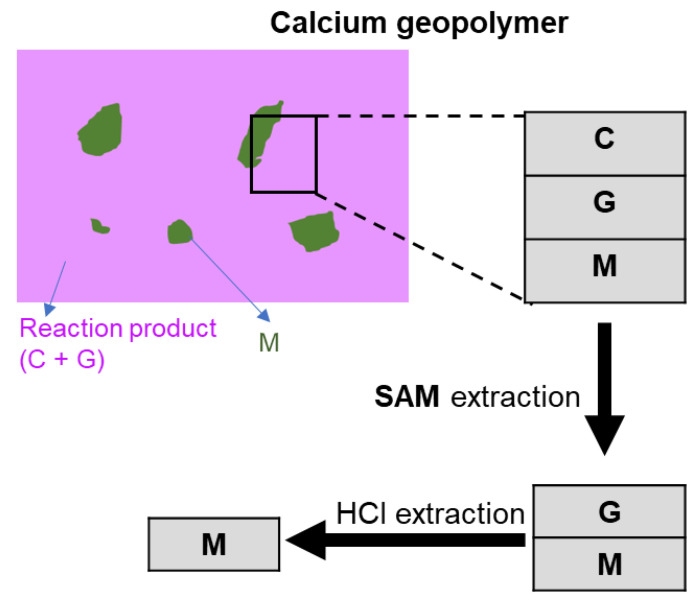
Flowchart of salicylic acid/methanol (SAM) and hydrochloric acid (HCl) extractions to selectively remove calcium silicate hydrate (C) and geopolymer gel (G) from calcium geopolymer that contains C, G and metakaolin (M).

Certain chemical extractions were performed to aid in assigning NMR peaks. For non-Ca mixtures, HCl extractions were conducted to remove geopolymer gel [32,33], using 1:20 volume ratio (i.e., 1 part 37 wt % HCl and 20 parts H_2_O). For Ca-mixtures, salicylic acid (HOC_6_H_4_COOH) in methanol (SAM) extractions [32,34,35,36] were carried out to remove the C-A-S-H and then HCl extractions used to remove geopolymer gel, as illustrated in Figure 1.

## 3. Results

### 3.1. Non-Ca Mixtures

#### 3.1.1. Degree of Reaction and Gel Composition

**Figure 2:**
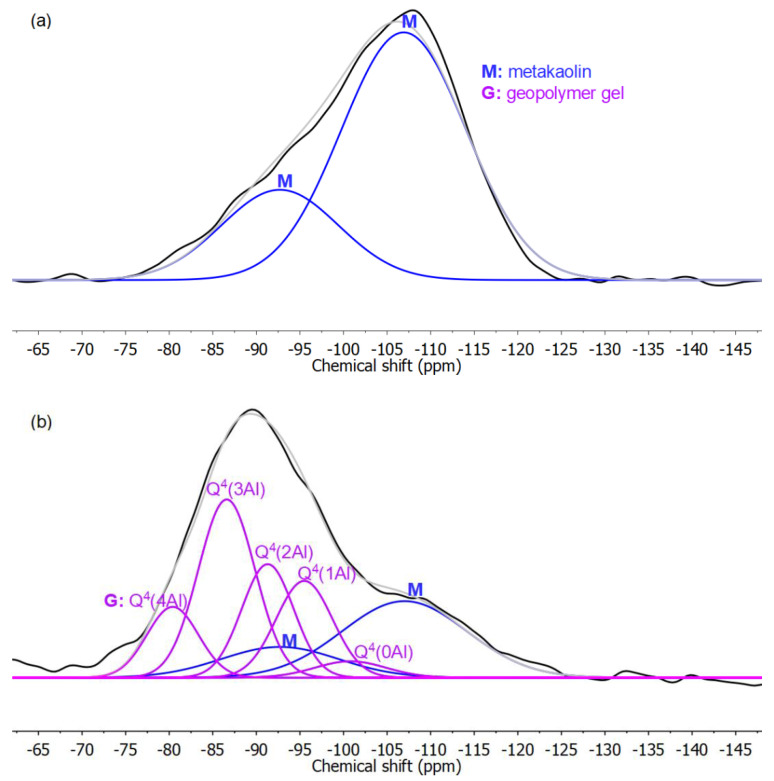
Deconvolution of the ^29^Si NMR spectra of (**a**) HCl residue and (**b**) specimen before chemical extractions for Mixture 4 with Si/Al of 1.5. The sum of deconvoluted peaks (silver) is close to the experimental spectrum (black).

The solid-state ^29^Si NMR spectra of the non-Ca mixtures were deconvoluted and the peaks were assigned. Figure 2 shows typical spectra. In the HCl residue (which contains only unreacted metakaolin [15]), the two peaks, −93 ppm and −107 ppm, are assigned to unreacted metakaolin. In the unextracted sample, the two metakaolin peaks exhibited the same width and relative intensity as those in the spectrum of the HCl residue. The remaining peaks were assigned to the geopolymer gel. The widths and positions of these peaks were kept consistent with those observed in deconvolution of a mature geopolymer, with fine adjustments to obtain good fitting. The resulting computed and measured spectra agreed well, though some low-intensity minor peaks (e.g., those between −65 and −75 ppm) are neglected and can account for about 5% of the total intensity. These deconvoluted peaks assigned to geopolymer gel were further assigned to Q^4^(nAl) (*n* = 0~4) sites [15,37].

Based on these assignments, the amount of each phase in terms of Si mol% was estimated. The degree of reaction was computed as Si mol% in the product with respect to that in the entire sample (i.e., the product phases and unreacted metakaolin), as defined by Equation (1):

(1) $$Degree\;of\;reaction=\frac{Moles\;of\;Si\;in\;product\;phases}{Moles\;of\;total\;Si\;in\;sample}$$

**Figure 3:**
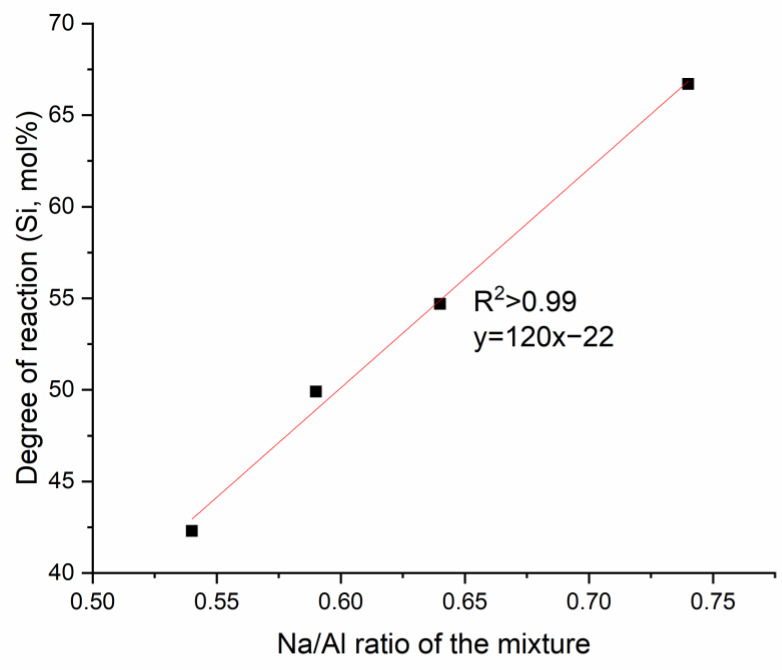
Degree of reaction from NMR analysis at different Na/Al ratios for the non-Ca mixtures.

The degree of reaction was found to be strongly linearly correlated with the Na/Al ratio of the mixture, as shown in Figure 3. However, as the relationship is based on only a few points, the degree of the correlation should not be overstated.

Also, based on the peak assignments, the Si/Al ratio of the geopolymer gel was estimated using Equation (2) [37]:

(2) $$Si/Al=\sum_{n=0}^{4}I_{Si\left(nAl\right)}/\sum_{n=0}^{4}\frac{n}{4}I_{Si\left(nAl\right)}$$

where *I_Si(nAl)_* is the intensity of each Q^4^(nAl) peak. Additionally, the Si/Al ratio of the unreacted metakaolin in these mixtures was calculated from the overall Si molar percent and the Si/Al ratio of geopolymer gel by considering that the summed Si/Al of these two phases is equal to the overall Si/Al ratio of the mixture. These results are summarized in Table 2.

#### 3.1.2. Relationships with Compressive Strength

**Figure 4:**
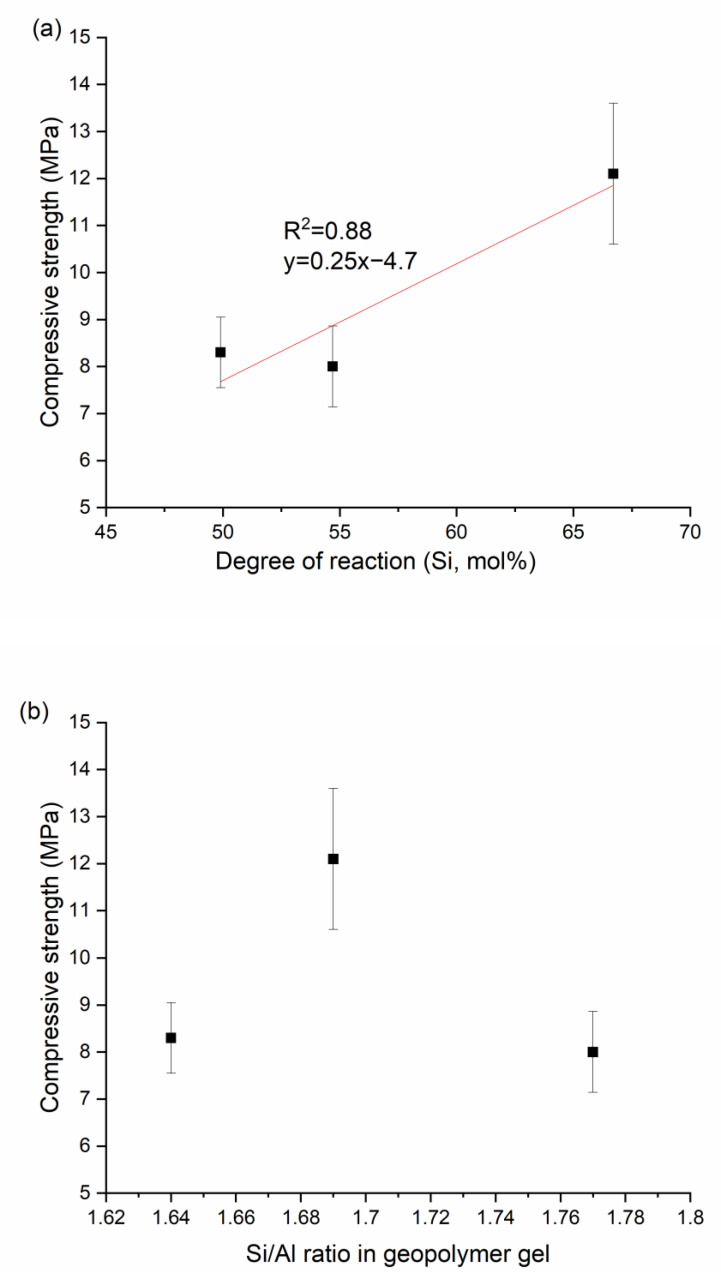
Compressive strength versus (**a**) degree of reaction and (**b**) Si/Al of geopolymer gel for non-Ca mixtures.

The 7-day compressive strength was plotted versus the degree of reaction (Si mol %) in Figure 4a. While the strength is generally low and error bars (half of the standard deviation of the mean on each side) are relatively high, the compressive strength increased, in a more or less linear manner, as the degree of reaction increased. While the R^2^ for the fitted equation y=0.25x−4.7 was found to be 0.88, this correlation is based only on limited data. Additional data points would provide more confidence in this relationship.

The strength was plotted versus the Si/Al ratio of the geopolymer gel in Figure 4b. The strength increased as Si/Al ratio changed from 1.64 to 1.69 and then decreased as the ratio increased further. These results do not reliably indicate a correlation between strength and Si/Al, mainly because the change in Si/Al ratio is extremely small. However, it is known that maximum strength usually occurs at an intermediate Si/Al ratio [12], typically around 1.90, a higher value than those observed here. It should be noted that Mixture 1 was excluded in both figures, as the samples were poorly consolidated as a result of extremely low workability. The strength of this mixture was considered unreliable for comparison. The low workability is likely related to the rapid geopolymerization due to the low Si/Al (1.1), which accelerates geopolymerization, and the low alkalinity (H_2_O/Na_2_O = 20), which minimizes any re-dissolution of gels [38,39].

Thus, it is seen that strength increased linearly with increasing degree of reaction and independently of gel composition. In general, degree of reaction and Si/Al of the geopolymer gel are linked in silicate-activated geopolymers, and their effects on strength have not been separated in previous studies [29]. Unlike earlier studies, in the current study we successfully separated the degree of reaction from the gel composition and so could explore its correlation with strength.

### 3.2. Ca Mixtures

#### 3.2.1. Degree of Reaction and Gel Composition

**Figure 5:**
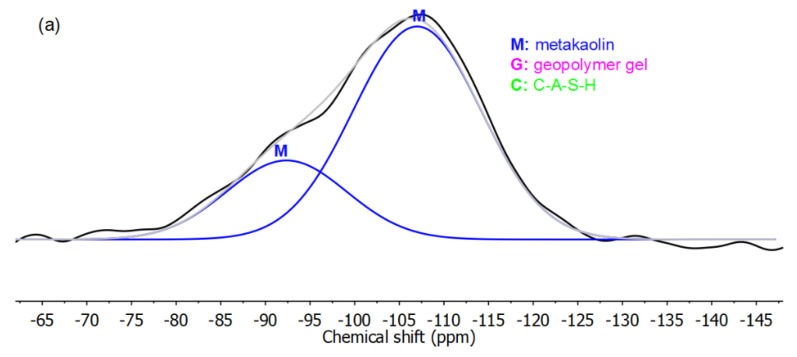

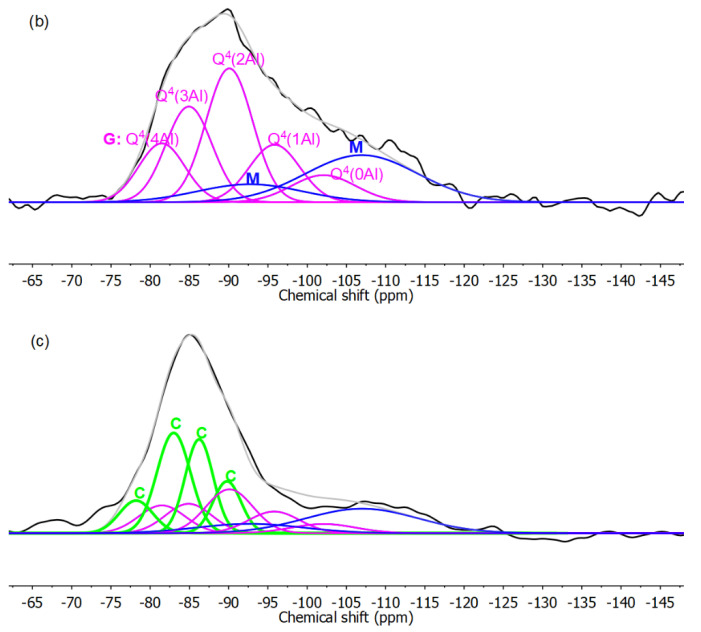
Deconvolution of the ^29^Si NMR spectra of (**a**) HCl residue, (**b**) SAM residue, and (**c**) specimen before chemical extractions for the Ca-mixture with Ca/Si of 0.15 and Si/Al of 1.5. The sum of deconvoluted peaks (silver) is close to the experimental spectrum (black).

The ^29^Si NMR spectra for mixtures containing calcium were deconvoluted as shown in Figure 5 for the solvent-exchanged mixture with Ca/Si 0.15 and its chemical extraction residues. The solvent-exchanged residue was extracted using SAM to remove the C-A-S-H, and the resulting residue was further extracted using HCl to remove the geopolymer gel, as detailed in Figure 1. To begin, the spectrum of the HCl residue was deconvoluted to two peaks, which were both assigned to metakaolin, expected to be the only phase remaining after the extractions [15]. In the spectrum of the SAM residue, peaks with the same width, position, and relative intensity were assigned to the unreacted metakaolin, and the remaining peaks were assigned to geopolymer gel. These gel peaks were similar to those assigned to geopolymer gel in the non-Ca specimens, with similar widths and positions. Additional peaks in the unextracted residue were assigned to C-A-S-H, according to spectra of synthesized C-A-S-H [40]. When these peaks were introduced to the deconvolution, with fine adjustments to achieve good fitting, the computed and measured spectra agreed well.

Based on these deconvolutions, the amount and the composition of each phase were analyzed. The molar percentages of C-A-S-H, geopolymer gel, and metakaolin were estimated to be 45.8%, 34.2%, and 20.0%, respectively. The five geopolymer peaks were assigned to Q^4^(nAl) (*n* = 0~4) sites, as noted in Figure 5b. The Si/Al of the geopolymer gel was calculated to be 1.85, somewhat higher than the 1.61 estimated for the corresponding non-Ca mixture (i.e., Mixture 4), a difference likely resulting from formation of a calcium modified gel that was extracted during the SAM treatment. By comparing the Si/Al of the gel with the bulk ratio, the Si/Al in the C-A-S-H was estimated to be 1.50. This ratio is lower than 5.0, the lower boundary of Si/Al observed in conventional C-A-S-H gel with chain structures [41], but it is within the range 1.2 to 10.0 for a calcium modified geopolymer gel, i.e., (Ca, Na)-A-S-H, with a three-dimensional structure [42], suggesting this SAM-extracted phase could be a calcium-modified geopolymer gel. This conclusion could not be drawn directly from peak positions in the spectrum in Figure 5c, because the Q^4^ peaks (associated with three-dimensional structures), if any, would overlap with the low-Q peaks (associated with chain structures) [43]. At any rate, such a calcium-modified geopolymer gel, if present, appears to have been dissolved together with any conventional C-A-S-H with chain structures during the SAM extraction. Such dissolution would explain the difference in the estimated Si/Al of the geopolymer gel between the Ca and the non-Ca mixtures noted above. To avoid such SAM-extraction related uncertainties, the degree of reaction for Ca-mixtures was examined below by a direct deconvolution of NMR spectra that separates unreacted metakaolin versus reaction products (i.e., C-A-S-H and geopolymer gels).

**Figure 6:**
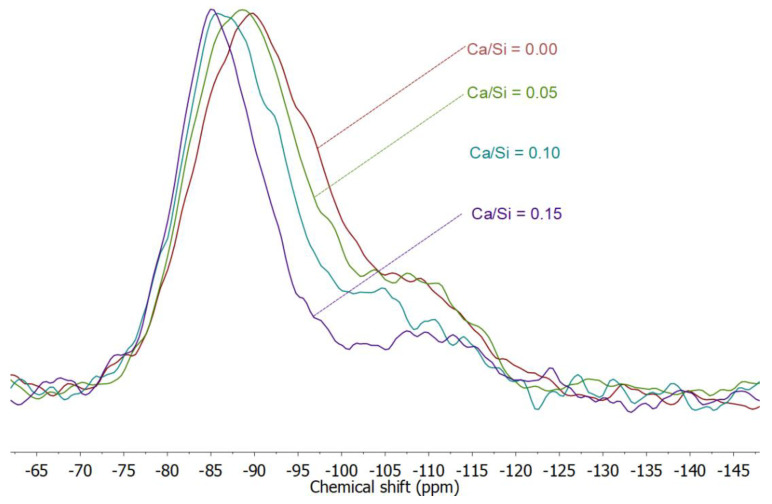
^29^Si NMR spectra of geopolymers at 7 days with different amounts of Ca.

With varying Ca amounts, the degree of reaction varied. Figure 6 shows the superimposed ^29^Si NMR spectra of the geopolymer specimens with different Ca amounts. The peak at about −107 ppm is in each case assigned to metakaolin. In this figure, the relative intensity in this region decreased as Ca/Si increased, indicating less unreacted metakaolin with higher Ca amounts.

**Figure 7:**
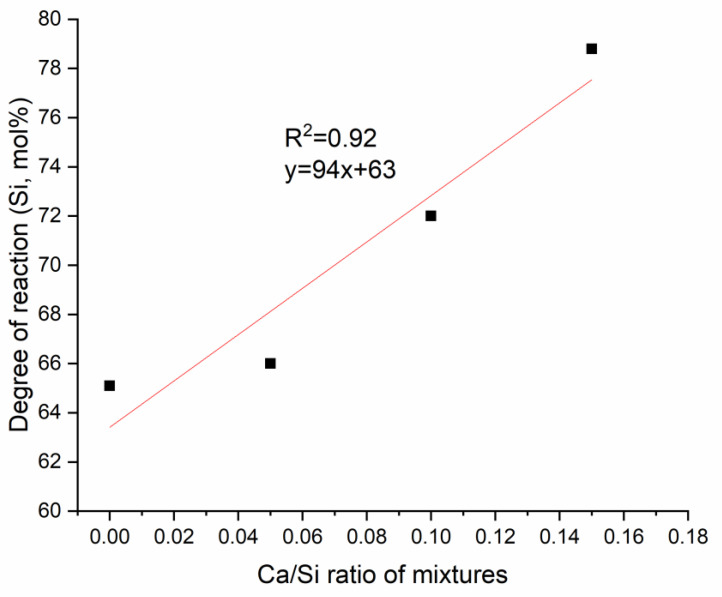
Degree of reaction from ^29^Si NMR analysis for geopolymers with different Ca/Si ratios at 7 days.

In order to quantify the observations, the unreacted metakaolin in terms of molar percentage of Si was calculated by estimating the intensity of the two metakaolin peaks relative to each whole spectrum (Figure 7). The degree of reaction was enhanced with Ca (at 7 days, as clearly seen in Figure 6 and quantified in Figure 7) and is consistent with the early-age results during geopolymerization [15,21]. As discussed in our previous study [15], and supported by other studies [44,45], at higher Si concentration, Al released from metakaolin would be more likely to contact and condense with Si species to form a precipitate on the surface of metakaolin particles that limits further dissolution. Because Ca reduces the concentration of Si, this surface precipitate is less likely to form when calcium is present.

#### 3.2.2. Relationships with Compressive Strength

**Figure 8:**
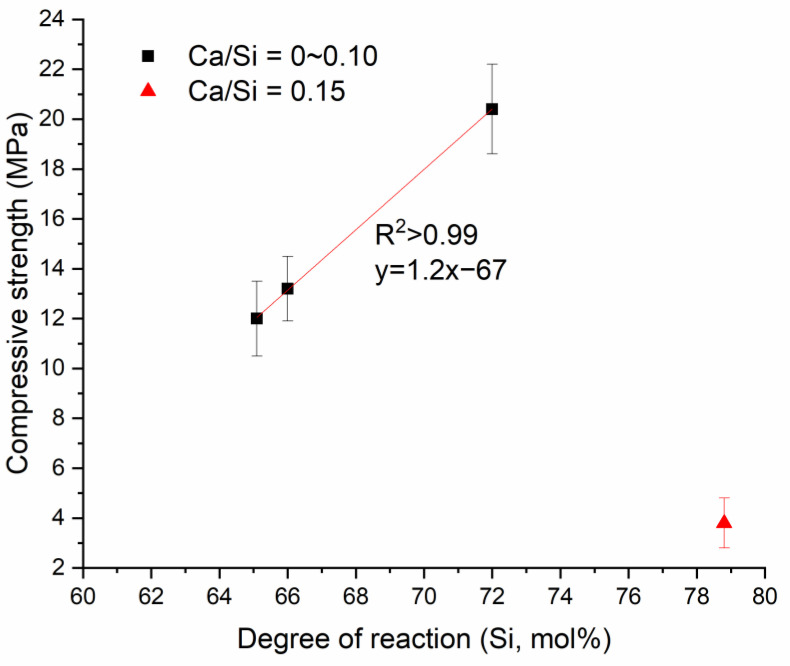
Compressive strength versus degree of reaction for geopolymers with different Ca/Si ratios at 7 days.

With Ca there are two reaction products, geopolymer gel and C-A-S-H, both of which are expected to contribute to strength, though not necessarily to the same degree. Compressive strength was plotted versus the degree of reaction for mixtures with different amounts of Ca in Figure 8. The geopolymer strength is substantially increased with addition of calcium, likely reflecting the enhanced degree of reaction with addition of calcium (see Figure 7). Mixtures with Ca/Si 0.10 and lower show a strong linear relationship between compressive strength and the amount of unreacted metakaolin. The correlation coefficient (R^2^) for this relationship is greater than 0.99, higher than that for the non-Ca mixtures above. It should be noted that the relationships are based only on limited data. The mixture with Ca/Si of 0.15 showed a low workability, which is believed to cause its extremely low compressive strength, so it was not included in the regression analysis. The low workability was associated with very rapid hardening, a phenomenon consistent with our and others’ previous studies [14,15,21].

One thing to be noted is that the slope of strength versus degree of reaction was much higher for the calcium mixtures (i.e., 1.2) in Figure 8 than for the non-calcium mixtures (i.e., 0.25) in Figure 4. Such difference suggests the presence of calcium-rich gel (i.e., C-A-S-H and calcium-modified geopolymer gel) produced strength more efficiently than the non-calcium geopolymer gel. This higher efficiency could be attributed to a higher Si/Al ratio of the geopolymer gel induced by calcium, as discussed in Section 3.2.1, since a higher Si/Al ratio of the gel generally exhibits a higher strength [12]. Additionally, the porosity could be reduced when the geopolymer gel and calcium-rich gel coexist [20]. Another observation that can be made from Figure 4a and Figure 8 is that the best-fit lines do not pass through the origin, likely reflecting a different structure-strength relationship when the degree of reaction is extremely low.

## 4. Conclusions

This study quantified the relationship between degree of reaction and strength for metakaolin geopolymers with and without Ca, independently of, for the first time, the effects of the gel Si/Al ratio. Degree of reaction was quantified using solid-state ^29^Si NMR. The increase of Na/Al ratio from 0.54 to 0.74 in the geopolymer gel increased the degree of reaction by >20%. Ca in the mixture (up to Ca/Si of 0.15) increased the degree of reaction by up to around 15%. For both non-Ca and Ca mixtures, the degree of reaction was correlated with the strength, with R^2^ > 0.88 for a linear relationship. Additionally, the combination of C-A-S-H and calcium-modified gel was found to produce strength more efficiently than mixtures containing only non-calcium geopolymer gel, as the curves of strength versus degree of reaction exhibited a higher slope for the calcium mixtures (i.e., 1.2) compared to the non-calcium mixtures (i.e., 0.25).

## Figures and Tables

**Table 1:** Compositions of mixtures

Mixture Number	Na/Al	Si/Al	H_2_O/Na_2_O	Ca/Si
1	0.54	1.10	20	0
2	0.59	1.20	20	0
3	0.64	1.30	20	0
4	0.74	1.50	20	0
5	0.74	1.50	20	0.05
6	0.74	1.50	20	0.10
7	0.74	1.50	20	0.15

**Table 2:** Amount and composition of geopolymer gel and unreacted metakaolin in the non-Ca mixtures as determined using NMR.

Mix No.	Mixture Design		Geopolymer Gel		Unreacted Metakaolin	
	Na/Al	Si/Al	Si/Al	Si, mol%	Si/Al	Si, mol%
1	0.54	1.1	1.4	42.3	0.95	57.7
2	0.59	1.2	1.64	49.9	0.95	50.1
3	0.64	1.3	1.77	54.7	0.98	45.3
4	0.74	1.5	1.69	65.1	1.22	34.9

## References

[1] Provis J.L., Palomo A., Shi C. (2015). Advances in understanding alkali-activated materials. Cem. Concr. Res..

[2] Duxson P., Fernández-Jiménez A., Provis J.L., Lukey G.C., Palomo Á., Van Deventer J.S. (2007). Geopolymer technology: The current state of the art. J. Mater. Sci..

[3] Provis J.L. (2018). Alkali-activated materials. Cem. Concr. Res..

[4] Palomo A., Grutzeck M., Blanco M. (1999). Alkali-activated fly ashes: A cement for the future. Cem. Concr. Res..

[5] Zhu W., Chen X., Struble L.J., Yang E.-H. (2019). Quantitative characterization of aluminosilicate gels in alkali-activated incineration bottom ash through sequential chemical extractions and deconvoluted nuclear magnetic resonance spectra. Cem. Concr. Compos..

[6] Bakharev T. (2005). Resistance of geopolymer materials to acid attack. Cem. Concr. Res..

[7] Suraneni P., Puligilla S., Kim E.H., Chen X., Struble L.J., Mondal P. (2014). Monitoring Setting of Geopolymers. Adv. Civ. Eng. Mater..

[8] Duxson P., Provis J.L., Lukey G.C., van Deventer J.S.J. (2007). The role of inorganic polymer technology in the development of ‘green concrete’. Cem. Concr. Res..

[9] Van Jaarsveld J., Van Deventer J., Lorenzen L. (1997). The potential use of geopolymeric materials to immobilise toxic metals: Part I. Theory and applications. Miner. Eng..

[10] Kriven W.M., Bell J.L., Gordon M. (2012). Microstructure and Microchemistry of Fully-Reacted Geopolymers and Geopolymer Matrix Composites. Ceramic Transactions Series.

[11] Chen X., Sutrisno A., Zhu L., Struble L.J. (2017). Setting and nanostructural evolution of metakaolin geopolymer. J. Am. Ceram. Soc..

[12] Duxson P., Provis J.L., Lukey G.C., Mallicoat S.W., Kriven W.M., Van Deventer J.S. (2005). Understanding the relationship between geopolymer composition, microstructure and mechanical properties. Colloids Surf. A Physicochem. Eng. Asp..

[13] Duxson P., Lukey G.C., Separovic F., Van Deventer J.S.J. (2005). Effect of Alkali Cations on Aluminum Incorporation in Geopolymeric Gels. Ind. Eng. Chem. Res..

[14] Yip C.K., Van Deventer J.S. (2003). Microanalysis of calcium silicate hydrate gel formed within a geopolymeric binder. J. Mater. Sci..

[15] Chen X., Sutrisno A., Struble L.J. (2018). Effects of calcium on setting mechanism of metakaolin-based geopolymer. J. Am. Ceram. Soc..

[16] Puligilla S., Chen X., Mondal P. (2019). Does synthesized C-S-H seed promote nucleation in alkali activated fly ash-slag geopolymer binder?. Mater. Struct..

[17] Puligilla S., Mondal P. (2015). Co-existence of aluminosilicate and calcium silicate gel characterized through selective dissolution and FTIR spectral subtraction. Cem. Concr. Res..

[18] Walkley B., Ke X., Hussein O.H., Bernal S.A., Provis J.L. (2020). Incorporation of strontium and calcium in geopolymer gels. J. Hazard. Mater..

[19] Li C., Sun H., Li L. (2010). A review: The comparison between alkali-activated slag (Si+Ca) and metakaolin (Si+Al) cements. Cem. Concr. Res..

[20] Yip C., Lukey G., Van Deventer J. (2005). The coexistence of geopolymeric gel and calcium silicate hydrate at the early stage of alkaline activation. Cem. Concr. Res..

[21] Temuujin J., Van Riessen A., Williams R. (2009). Influence of calcium compounds on the mechanical properties of fly ash geopolymer pastes. J. Hazard. Mater..

[22] Nedeljković M., Li Z., Ye G. (2018). Setting, Strength, and Autogenous Shrinkage of Alkali-Activated Fly Ash and Slag Pastes: Effect of Slag Content. Materials.

[23] Boumiz A. (1996). Mechanical Properties of Cement Pastes and Mortars at Early Ages Evolution with Time and Degree of Hydration. Adv. Cem. Based Mater..

[24] De Schutter G., Taerwe L. (1996). Degree of hydration-based description of mechanical properties of early age concrete. Mater. Struct..

[25] Luna-Galiano Y., Fernández-Pereira C., Izquierdo M. (2016). Contributions to the study of porosity in fly ash-based geopolymers. Relationship between degree of reaction, porosity and compressive strength. Mater. Constr..

[26] Kumar P., Pankar C., Manish D., Santhi A. (2018). Study of mechanical and microstructural properties of geopolymer concrete with GGBS and Metakaolin. Mater. Today Proc..

[27] Duxson P., Provis J.L., Lukey G.C., Separovic F., Van Deventer J.S.J. (2005). 29Si NMR Study of Structural Ordering in Aluminosilicate Geopolymer Gels. Langmuir.

[28] Kim E.H. (2012). Understanding Effects of Silicon/Aluminum Ratio and Calcium Hydroxide on Chemical Composition, Nanostructure and Compressive Strength for Metakaolin Geopolymers.

[29] Williams R.P., Hart R.D., Van Riessen A. (2011). Quantification of the Extent of Reaction of Metakaolin-Based Geopolymers Using X-ray Diffraction, Scanning Electron Microscopy, and Energy-Dispersive Spectroscopy. J. Am. Ceram. Soc..

[30] ASTM (2016). Standard Test Method for Compressive Strength of Hydraulic Cement Mortars.

[31] Chen X., Meawad A., Struble L.J. (2014). Method to Stop Geopolymer Reaction. J. Am. Ceram. Soc..

[32] Granizo M.L., Alonso S., Blanco-Varela M.T., Palomo A. (2004). Alkaline Activation of Metakaolin: Effect of Calcium Hydroxide in the Products of Reaction. J. Am. Ceram. Soc..

[33] Palomo Á., Alonso S., Fernandez-Jiménez A., Sobrados I., Sanz J. (2004). Alkaline Activation of Fly Ashes: NMR Study of the Reaction Products. J. Am. Ceram. Soc..

[34] Struble L. (1985). The effect of water on maleic acid and salicylic acid extractions. Cem. Concr. Res..

[35] Stutzman P.E. (1996). Guide for X-ray Powder Diffraction Analysis of Portland Cement and Clinker.

[36] Alonso S., Palomo Á. (2001). Calorimetric study of alkaline activation of calcium hydroxide–metakaolin solid mixtures. Cem. Concr. Res..

[37] Engelhardt G., Michel D. (1987). High Resolution Solid State NMR of Silicates and Zeolites.

[38] Chen X., Mondal P. (2020). Effects of NaOH amount on condensation mechanism to form aluminosilicate, case study of geopolymer gel synthesized via sol–gel method. J. Sol-Gel Sci. Technol..

[39] Zhang Z., Wang H., Provis J.L., Bullen F., Reid A., Zhu Y. (2012). Quantitative kinetic and structural analysis of geopolymers. Part 1. The activation of metakaolin with sodium hydroxide. Thermochim. Acta.

[40] Hunnicutt W.A. (2013). Characterization of Calcium-Silicate-Hydrate and Calcium-Alumino-Silicate-Hydrate.

[41] Myers R.J., L’Hôpital E., Provis J.L., Lothenbach B. (2015). Effect of temperature and aluminium on calcium (alumino)silicate hydrate chemistry under equilibrium conditions. Cem. Concr. Res..

[42] García-Lodeiro I., Palomo Á., Fernández-Jiménez A., Macphee D.E. (2011). Compatibility studies between N-A-S-H and C-A-S-H gels. Study in the ternary diagram Na_2_O–CaO–Al_2_O_3_–SiO_2_–H_2_O. Cem. Concr. Res..

[43] Walkley B., Provis J.L. (2019). Solid-state nuclear magnetic resonance spectroscopy of cements. Mater. Today Adv..

[44] Favier A., Habert G., De Lacaillerie J.-B.D., Roussel N. (2013). Mechanical properties and compositional heterogeneities of fresh geopolymer pastes. Cem. Concr. Res..

[45] Provis J.L., Lukey A.G.C., Van Deventer J.S.J. (2005). Do Geopolymers Actually Contain Nanocrystalline Zeolites? A Reexamination of Existing Results. Chem. Mater..

